# One type of graft for reconstruction of the ACL does not suit all patients based on their characteristics and sports: a scoping review

**DOI:** 10.1007/s12306-024-00861-x

**Published:** 2024-09-09

**Authors:** J. M. Reinerink, T. Vendrig, M. N. J. Keizer, R. A. G. Hoogeslag, R. W. Brouwer

**Affiliations:** 1 Center for Human Movement Sciences, University of Groningen, University Medical Center Groningen, UMCG Sector F, FA 23,Antonius Deusinglaan 1, PO Box 317, 9713 AV Groningen, The Netherlands; 2 Centre for Orthopaedic Surgery and Sports Medicine OCON, Hengelo, The Netherlands; 3 Department of Orthopedic Surgery, Martini Hospital Groningen, Groningen, The Netherlands

**Keywords:** Patient characteristics, Graft selection, Age, Gender, Body height, Type and level of sports activity

## Abstract

The selection of graft type for anterior cruciate ligament reconstruction remains a topic of debate, taking into consideration patient characteristics, as well as the type and level of sports involvement. The aim of this scoping review was to investigate patient characteristics that might influence the selection of graft type for anterior cruciate ligament reconstruction. PubMed and Scopus were searched to identify articles for inclusion. All included studies focused on one or more patient characteristics involved in the decision-making process regarding anterior cruciate ligament reconstruction autograft, including the hamstrings tendon (HT), patellar tendon (BPTB) and quadriceps tendon (QT). Out of the 1,977 initial studies, 27 studies were included in this review. The BPTB graft seems to be the preferred choice in young patients, females, and athletes—especially those engaged in pivoting sports. The HT graft seems to be the preferred choice in less active and older patients, along with those involved in sports where knee extensors are vital. The HT graft is not preferable in patients with a small body height and graft diameter. Moreover, surgeon preferences were also of importance for graft selection. The success of a specific graft type in anterior cruciate ligament reconstruction is highly dependent on the patient’s characteristics and type of sport. Patient characteristics such as age, gender, body height, graft diameter, and the patient’s activity level should all be considered when choosing the appropriate graft type.

## Introduction

Historically, the first extra-articular procedures to treat anterior cruciate ligament (ACL) injuries were reported by Bennett in 1926, and Cotton, Morrison, and Bosworth in the mid-1930s. Campbell described the first intra-articular procedure for ACL reconstruction (ACLR) using a bone-patellar tendon-bone (BPTB) graft [1]. The trends of graft selection for intra-articular reconstruction have evolved through four phases: beginning with BPTB autograft dominance, followed by increasing use of hamstring tendon (HT) autograft alongside BPTB autograft dominance, and then a shift towards HT autograft dominance with fewer BPTB autograft and the emerge of allografts, and lastly, HT autograft dominance with steady BPTB autograft usage and the emergence of quadriceps tendon (QT) autograft usage [2]. Nowadays, there is still an expanding body of research in the field of ACLR [3], as well as advancements in surgical techniques knowledge. However, despite these developments, many athletes fail to return to their pre-injury sports level, and up to 30% of the patients re-rupture their ACL. These outcomes may be due to clinical failures caused by sport activities, changed neuromechanics or clinical failure of the graft type [4]. Consequently, the selection of graft for ACLR and the influence of patients’ characteristics as well as sports type are subjects of ongoing debate [3].

An ACLR is especially important for patients aiming to return to sport (RTS) [5]. The primary goal of ACLR is to restore knee anatomy, stability, and function while preventing post-traumatic osteoarthritis [6]. Each type of autografts has its advantages and disadvantages, with none considered ‘ideal’ [7]. For example, HT autograft exhibits good clinical outcomes but may lead to knee flexor muscle weakness [8] and show individual variability in the tendon diameter [9]. BPTB offers good graft stability due to bone-to-bone fixation but can result in donor site morbidity, especially anterior knee pain [8]. QT autografts seem to have advantages, including reduced harvest morbidity and superior structural properties [10], although limited evidence supports the overall benefit of the QT autograft [10]. It is, however, shown that graft selection and surgical techniques are influenced by a variety of factors, including surgeon preference based on patient’s characteristics or experience and patient preference [11].

Within the literature, there are numerous patient’s characteristics and sport types mentioned that possibly have an influence on the graft selection, often without clear explanation and compelling arguments. Therefore, there is a need for a clear overview of factors influencing surgeons’ decisions, which focuses on patient characteristics and type of sports. This scoping review focuses solely on graft selection for autografts. The authors aim to provide insight into what is known about indicators that influence autograft graft selection, using the best available evidence. By doing so, the authors endeavour to identify related factors to patient characteristics, type and level of sports, and their impact on graft selection decision-making.

## Methods

### Study design

Due to the exploratory and descriptive nature of the research question, a scoping literature review was conducted using terms related to patient characteristics influencing the graft selection in ACLR. The steps outlined by Arksey and O’Mallay were followed for this review [12]. These steps included the following five steps: 1) the research question was clarified and the purpose of the study was defined; 2) the relevant studies were identified by balancing feasibility with breadth and comprehensiveness; 3) studies using an iterative team approach were selected to study selection and data extraction; 4) the data were charted; 5) the results were collected, summarized and reported.

### Search strategy

Literature was searched in the electronic databases PubMed and Scopus on March 8, 2024. Terms for the database searches included ‘anterior cruciate ligament reconstruction’ or ‘ACLR’, ‘patient characteristics’ and ‘graft selection’. The full search strategy can be found in the appendix. First the title and abstract were screened by the first author (JMR). Two authors (JMR, TV) independently reviewed the full texts of the identified studies. There was disagreement on two articles; this was resolved by consensus.

### Inclusion and exclusion criteria

Only articles written in English were included in this study. The publication date was limited to the past 7 years since the search date, considering the relevance and the volume of research conducted in this field, as well as the change in surgical techniques used over the past decade. Only articles focusing on one or more patient characteristics in two or more autografts were included in this study. Articles focusing solely on the surgical technique being used in the different grafts, multi-ligamentous injuries, allografts, as well as comparisons between allografts and autografts, were excluded from this study. Articles including patients with open physes were excluded because the decision-making for ACLR in patients with open physes is substantially different as there is no consensus surrounding the grey area of skeletal maturity [13, 14]. When relevant information was identified in a systematic review, the concerning articles were examined and included when they met the inclusion criteria.

### Data extraction and charting

Data extraction, performed by the first author (JMR), included author, year, type of study, intervention type and comparator when existing, duration of the intervention, study population, aims of the study, methodology, outcome measures, significant results, and considerations for future research. The studies were grouped by the highlighted patient characteristics in the article, and extracted data were summarized.

## Results

**Fig. 1:**
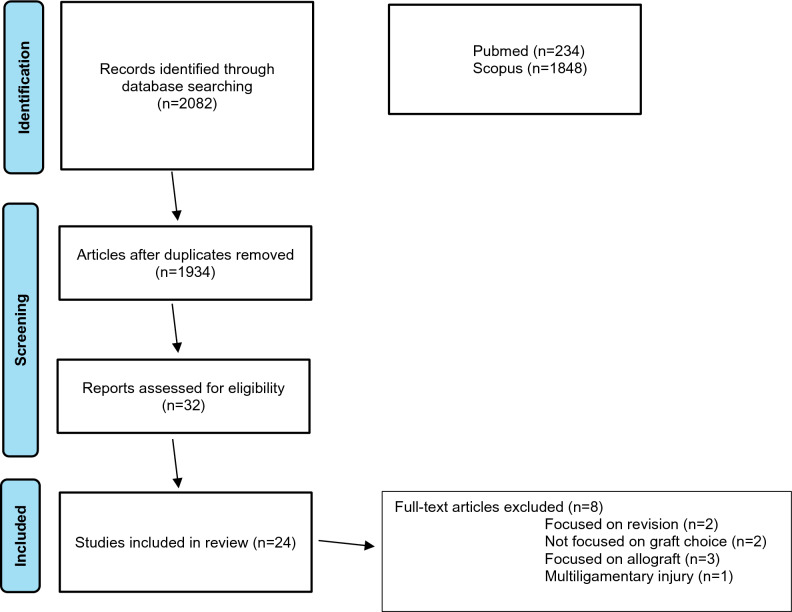
Flow diagram of the study selection procedure

Thirty-two studies were initially selected for the data extraction process. This full-text screening resulted in the exclusion of seven articles that did not meet the previously mentioned inclusion criteria. Eventually, data were extracted from 24 articles (see Fig. 1).

In Table 1, the characteristics, as well as the level of evidence, of the included studies are listed.

**Table 1:** Characteristics of the included studies

Study	Type of study	Grafts	Characteristics	Number of participants
Bowman et al. [16]	Cross-sectional study	BPTB, HT, QT	Surgeon preference based on age and sports	514
Bowman et al. [16]	Narrative review	BPTB, HT, QT	Patient age, gender, graft diameter, type and level of sports activity	
Etzel et al. 2022 [18]	Systematic review	BPTB, HT	Patient age, gender	1385 (total), 655 BPTB, 525 HT
Herman et al. 2023 [28]	Combined retrospective study	BPTB, QT	Type and level of sports activity	23 BPTB, 14 QT
Lin et al. [17]	Review	BPTB, HT, QT	Patient age	N.m
Spindler et al. [25]	Cohort study	BPTB, HT	Patient age, type and level of sports activity	770 (total), 492 BPTB, 278 HT
Salem et al. [42]	Cohort study	BPTB, HT	Patient age	256 (total), 175 BPTB, 81 HT
Maletis et al. [19]	Review?	BPTB, HT	Patient age	36,186
Svantesson et al. [22]	Systematic review	BPTB, HT	Gender	N.m
Alomar et al. [33]	Systematic review and meta-analysis	HT	Graft diameter	19,799
Kyung et al. [37]	Editorial	BPTB, HT, QT	Graft diameter	N.m
Matzkin et al. [35]	Review	BPTB, HT	Graft diameter	N.m. (8 studies included)
Schwartzberg et al. [23]	Editorial	BPTB, HT, QT	Body height	N.m
Belk et al. [27]	Editorial	BPTB, HT	Type and level of sports activity	N.m
Britt et al. [26]	Case study	BPTB, HT	Type and level of sports activity	71
Arnold et al. [2]	Survey	BPTB, HT, QT	Type and level of sports activity, Surgeon	N.m
Lesevic et al. [14]	Cohort study	BPTB, HT	Gender, type and level of sports activity	166 (total), 100 BPTB, 66 HT
Shah et al. [21]	Case study	BPTB, HT	Patient age	51
Sollberger et al. (2022)	Systematic review	BPTB, HT	Type and level of sports activity	1833
Goto et al. [24]	Cohort study	QT	Body height	73
Musahl et al. [36]	Review	BPTB, HT, QT	Graft diameter	N.m
Baawa-Ameyaw et al. [15]	Instructional review	BPTB, HT, QT	Patient age, type and level of sports activity, Surgeon	N.m
Fischer et al. [29]	Cohort study	HT, QT	Type and level of sports activity	124
Martin-Alguacil et al. [30]	Randomized controlled trial	HT, QT	Type and level of sports activity	56
Moatshe et al. [31]	Review	BPTB, HT, QT	Surgeon	N.m
Cerciello et al. [32]	Survey	BPTB, HT, QT	Surgeon	140

BPTB, bone-patellar tendon-bone graft; HT, hamstring tendon graft; QT, quadriceps tendon graft; N.m., not mentioned

**Fig. 2:**
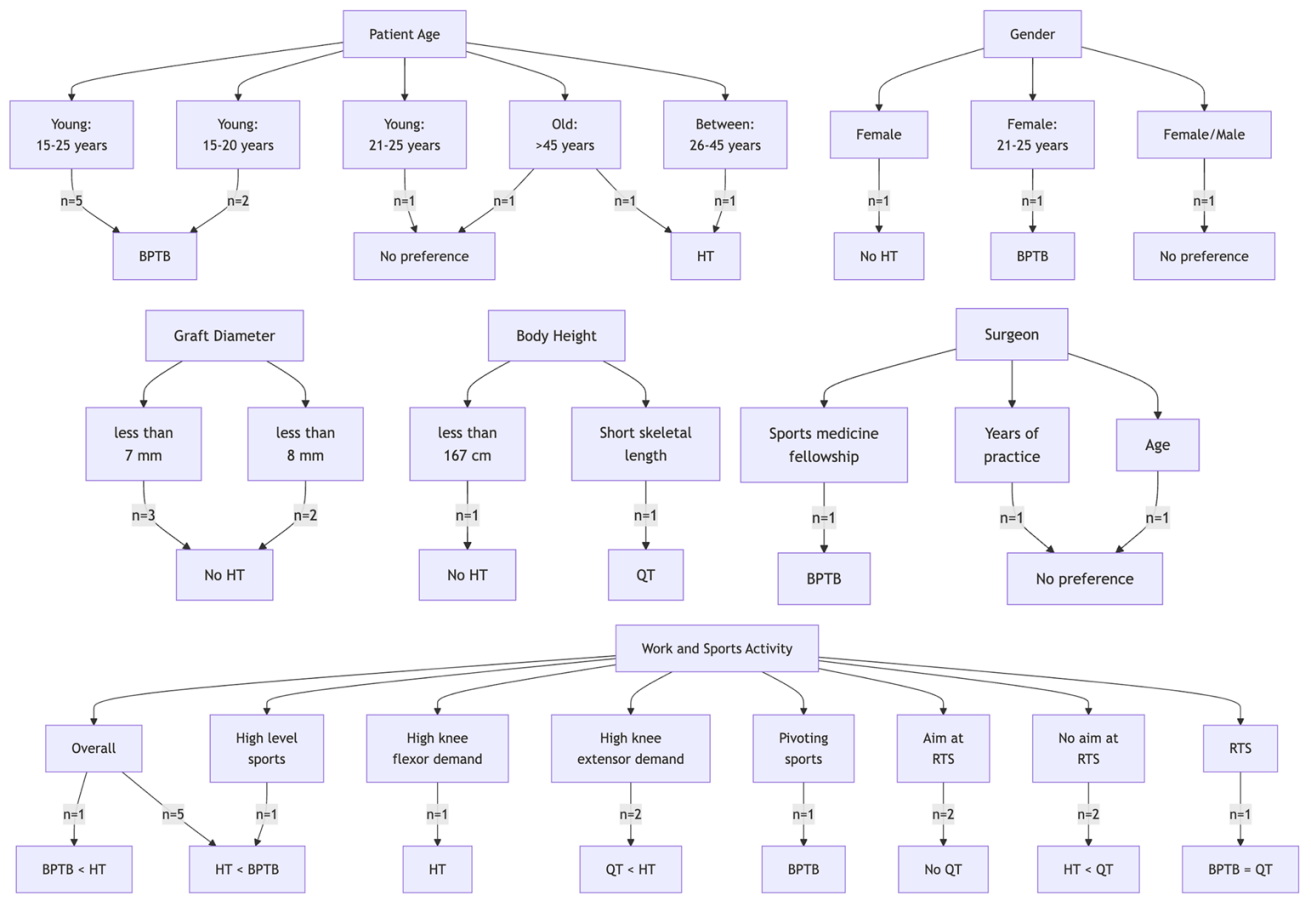
Summary flow chart of the results. < , less preferred than; = , same as; BPTB, bone-patellar tendon-bone graft; HT, hamstring tendon graft; QT, quadriceps tendon graft; RTS, return to sport

In Table 2, the most important patient characteristics are listed. Subsequently, all characteristics will be discussed in further detail. Patient characteristics are interrelated causing limited amount of overlap in the upcoming paragraphs. In Fig. 2, a summary flow chart of the results is presented.

**Table 2:** Patient characteristics and preferred graft per patient characteristic

Characteristic	Studies	Groups	Preferred graft
Patients age	Bowman et al., [16], De Petrillo et al., [13], Etzel et al., [18], Lin et al., [17], Spindler et al., [25]	Young (15-25y)	BPTB
	Salem et al., [42], Maletis et al., [19]	Young (15-20y)	BPTB
	Salem et al., [42]	Young (21-25y)	No preference
	Lin et al., [17]	Between (26-45y)	HT
	Lin et al., [17]	Old (>45y)	HT
	Shah et al., [21]	Old(>45y)	No preference
Gender	Svantesson et al., [22]	Female/male	No preference
	De Petrillo et al, [13]	Female	No HT
	Etzel et al, [18]	Female (21-25y)	BPTB
Graft diameter	De Petrillo et al., [13], Alomar et al., [33], Kyung et al., [37]	< 7 mm	No HT
	Matzkin et al., [35], Musahl et al., [36]	< 8 mm	No HT
Body height	Schwartzberg et al., [23]	< 167 cm	No HT
	Goto et al., [24]	Short skeletal lengths	QT
Work & type and level of sports activity	Belk et al., [27], De Petrillo et al., [13], Britt et al., [26], Baawa-Ameyaw et al., [15], Sollberger et al., 43	Overall	HT < BPTB
	Arnold et al., [2]	High level sports Overall	BPTB < HT
	Spindler et al., [25]		BPTB
	Bowman et al. [16]	Pivoting sports	BPTB
	De Petrillo et al., [13]	High knee flexor demand	HT
	De Petrillo et al., [13], Lesevic et al., [14]	High knee extensor demand	QT < HT
	Fischer et al., [29], Martin-Alguacil et al., [30]	Aim at RTS	No QT
	Fischer et al., [29], Martin-Alguacil et al., [30]	No aim at RTS	HT < QT
	Herman et al., [28]	RTS	BPTB = QT
Surgeon	Bowman et al., [16]	Sport medicine fellowship^1^	BPTB
	Bowman et al., [16]	Years in practice	No preference
	Bowman et al., [16]	Age	No preference

< , less preferred than; = , same as; BPTB, bone-patellar tendon-bone graft; HT, hamstring tendon graft; QT, quadriceps tendon graft; RTS, return to sport

^a^(one-year) academic fellowship in sports medicine

### Patient age

Age is seen as the most important patient characteristic to consider when deciding on an appropriate graft selection for ACLR [15–17]. Preferences for ACLR grafts in those patients are divided between ‘young’ (15 to 25 years) and ‘old’ (> 45 years), with some variation among articles. The most widely accepted graft type for young patients with closed physes is BPTB graft. The preference for BPTB graft is strengthened when these young patients are athletes or patients returning to high-risk activities [13, 16–20]. However, for older patients (> 45 years) a HT graft is preferred [17].

In the study of Lin et al. [17], age categories were not specified. However, for patients who are still physiologically young and moderately active, the HT graft is recommended [17]. The same recommendations are made for older and less active patients. The article by Shah et al. [21] found no differences regarding graft selection for patients over forty-five undergoing primary ACLR.

### Gender

Gender may be an important factor to consider when choosing the appropriate graft type for ACLR. While no hard evidence was found to support specific graft recommendations for gender, some information suggests potential differences. It is suggested that graft selection outcomes may differ between females and males due to several factors. Females are more likely to sustain an ACL rupture compared to males [22], there are anatomical differences between males and females, and male patients have a higher risk of re-rupture, quadriceps muscle strength imbalance, thicker tendon diameters, and a different knee flexor function [14, 22]. However, it is important to note that there is a gap in literature regarding graft selection recommendations for males and graft selection differences between males and females, and there is a need for more research in this area.

However, there is some evidence regarding the consideration of graft selection for females. One disadvantage in females is the generally smaller tendon diameter, which increases the risk of graft failure, especially in HT autografts [13]. Therefore, the use of a QT or BPTB graft may be more suitable in females. Furthermore, studies focusing on young females (≤ 25 years old) have shown a lower graft failure rate for BPTB autografts compared to HT autografts [13, 18]. This difference is especially significant for female patients aged between 15 to 20 years, where the use of a BPTB graft resulted in a significant lower failure rate compared to HT grafts [18]. Additionally, a natural imbalance in the quadriceps to hamstring muscle activation ratio was observed in females during activity [13]. Using a HT autograft could exacerbate this imbalance and enhance the risk of graft failure. When considering knee flexor function, gender should be taken into account considering the interaction between sex and graft type [14]. Female patients treated with a HT autograft showed lower knee flexor peak torque than females treated with a BPTB autograft, suggesting BPTB grafts may be a better choice than HT grafts for preserving knee flexor function. Overall, a tendon of the extensors may be superior for females over a tendon of the flexors.

### Body height

The patient’s height is related to the length and diameter of the semitendinosus tendon [23]. The problem concerning graft diameters is that a smaller graft diameter may increase graft failure rate [13]. In shorter patients, especially in shorter females, the length of the HT graft does not allow for a quadrupled graft construct of acceptable length. Therefore, the HT graft is discouraged in short patients [23]. Goto et al. [24] argues that QT graft is a viable option for shorter skeletal lengths with similar results for muscle strength recovery, morbidity, and readiness to RTS between the small-statured female group and the control group.

### Type and level of sports activity

Activity level is an important factor to consider when deciding on the ideal ACL graft for a patient. For patients participating in sports such as soccer, sprinters, and judo, a post-operative knee flexor deficit, potentially caused by HT graft harvest, could limit their performance [13]. In such a case, BPTB grafts could be the preferred choice [13]. Indeed, BPTB grafts show positive functional outcomes and good RTS [15, 25, 43] and are especially preferred in patients participating in pivoting sports [16]. Athletes competing in high school, club, or college sports had a higher chance of receiving a BPTB graft compared to patients participating at a recreational level [20]. Britt et al. [26] found that for female soccer players, BPTB grafts resulted in a higher proportion of patients returning to any level of soccer, as well as preinjury levels, compared to HT grafts. Moreover, they found that HT grafts failed 2 times more frequently and fear was reported twice as often in patients with a HT graft compared to the BPTB graft in female soccer players. However, they did not find significant results in RTS. On the contrary, Belk et al. [27] reported that BPTB grafts resulted in higher RTS and return to pre-injury level of sports. Moreover, they found lower rotational stability for BPTB grafts compared to HT grafts in athletes aged 15 to 25 years.

For sports such as volleyball or alpine skiing, knee extensors play a crucial role. They are essential for bilateral jumping and situation-dependent knee joint loading [13]. QT grafts or BPTB grafts might cause a post-operative knee extensor deficit and are therefore not preferred in patients performing these kinds of sports [13]. Also, when knee flexion function is decreased by choosing a HT graft over the BPTB graft, the quadriceps-to-hamstrings force ratio will shift towards the quadriceps, resulting in stronger quadriceps in relation to the hamstrings, which is suggested to be preferable in sports were knee extensors are important.

Herman et al. [28] found similar RTS results for BPTB grafts compared to QT grafts in female soccer players. Also, QT grafts showed, within one post-operative year, similar results to HT grafts in knee flexion torque and adequate strength levels, however, resulted in more knee extension strength deficit [29, 30]. When a patient aims for RTS where the quadriceps are important, this knee extension strength deficit should be considered and a graft of the extensors may not be ideal. If this is not the intention, the QT graft should be favoured over the HT graft, considering the quadriceps-to-hamstring muscle activation ratio, which is in favour of QT graft [29, 30]. In this case, the hamstrings remain stronger in relation to the quadriceps, which theoretically results in less forward pull on the tibia due to muscle activity.

### Work

Nowadays there is no existing literature regarding the type of work and graft selection; however, it may be important to investigate whether the job of the patient requires frequently kneeling activities. In such cases, BPTB may not be optimal due to the increased risk of anterior knee pain [2, 15].

### Surgeon

First, it should be noted that the surgeon ultimately has the largest influence on the graft used for ACLR [20, 31]. The familiarity of the surgeon with a specific graft selection and technique performed should be considered in the decision-making process [15, 32]. Bowman et al. [16] found that the number of years in practice and the age of the surgeon did not influence the selection of graft type. However, surgeons who completed a sports medicine fellowship were more inclined to choose BPTB graft over HT graft compared to surgeons who did not follow a sports medicine fellowship [16]. Altogether, the surgeon’s preference is of great importance in the decision-making process. However, an individualized approach that considers factors influencing the functional outcomes of ACLR is becoming increasingly important and should be taken into consideration as well [2, 31, 32].

## Discussion

This scoping review highlights the patient characteristics that influence the graft selection for ACLR, as identified in scientific literature. Most importantly, this review emphasizes the need for a personalized approach in graft decision-making, and orthopaedic surgeons treating ACL injured patients should be aware that a single technique may not be suitable for all patients. Factors such as age, gender, body height, graft diameter, and the patient’s activity level should all be considered when choosing the appropriate graft type. Moreover, the findings of this review uncovered several gaps in the literature, which will be discussed below. These gaps include issues with generalizability in studies, a lack of research surrounding upcoming graft types, such as the QT graft, and conflicting results in studies regarding specific characteristics, such as graft diameter and age.

An overall preference for the BPTB graft was found for younger patients, females, and athletes, especially patients participating in pivoting sports. This preference may be because of the lower failure rate and high chance to return to pre-injury level of sports associated with BPTB autografts. Conversely, an overall preference for the HT graft was found for moderately or less active and older patients and patients participating in sports where knee extensors play a crucial role, such as in skiing. This preference may be due to less donor site morbidity, lower knee pain, and higher residual quadriceps strength compared to the use of BPTB grafts. The most disagreement in the literature appears to concern young female athletes. Specifically, the BPTB graft has a lower re-rupture rate compared to the HT graft, but using a BPTB graft may result in more donor site morbidity, potentially leading to complications such as reduced strength and decreased range of motion. Moreover, the patient’s desired level and type of activity should be considered when deciding on the graft type, since harvesting a specific tendon may possibly lead to limitations in movements associated with that tendon.

Related to these patient characteristics, is graft diameter. The height of the patient is a predictor for the diameter of the semitendinosus tendon. The problem concerning graft diameters is that a smaller graft diameter may increase graft failure rate [13]. Moreover, a thin graft can lead to disadvantages of the healing process, which is of particular concern for women [13]. The diameter for a HT graft used for ACLR should be over 7 mm according to the reviews of Alomar et al. [33], Spragg et al. [20] and Magnussen et al. [34], and over 8 mm according to Matzkin et al. [35]. With this diameter thickness, the likelihood of graft failure decreases [35]. An advice to aim for larger diameters [13, 36], or a minimum of at least 9 mm [35], is given when ACLR involves patients < 20 years of age, female athletes, or patients participating in high-demanding sports. There may be evidence to use a QT graft because the minimum graft diameter mentioned above cannot be expected in every HT graft for all patients [23]. Overall, a HT graft causes more complications than the BPTB graft considering graft diameter [13, 35, 37]. Therefore, graft diameter is important in graft selection [13, 35, 36] and for this reason, especially in female, an extensor tendon may be the superior choice over a HT graft.

What this review does not address, but nowadays an important consideration for surgeons when making graft selections, is the use of additional procedures combined with ACLR using HT, BPTB, or QT graft. For instance, various surgical procedures aimed at stabilizing the anterolateral corner have been recently introduced, such as a lateral extra-articular tenodesis (LET) using the iliotibial band [38, 39]. This procedure appears to be beneficial for limiting rotational stability in ACLR patients and improving subjective outcomes [38, 39]. Moreover, a logistic regression model with the same predictors as discussed in the current review showed that patients who received HT alone were 3.4 times more likely to experience a re-rupture compared to those who received a combination of HT and a LET [40]. These results may confirm the protective nature of HT combined with LET against re-ruptures in young, active patients when compared to HT graft alone. This lower failure rate may be due to the ability of a LET to provide greater control of rotational laxity, as supported by multiple of biomechanical and clinical studies [39, 41].

In the context of the current literature on ACLR, this scoping review brings together an extensive body of recent research to emphasize crucial factors in graft selection. The trends observed across diverse studies underscores the crucial impact of the patients age, graft diameter, and the surgeon. The results provide a nuanced understanding of the dichotomy in choices for ‘young’ and ‘older’ individuals, as well as clear recommendations for sports were knee flexors are of great importance [13, 16–20, 42]. However, while this review captures the prevailing trends in ACLR, it also uncovers notable gaps. Specifically, there is limited evidence on ACLR recommendations for the middle-aged population, males, and populations engaged in specific jobs. Also, a notable gap exists surrounding the recommendations for the use of QT grafts, emphasizing the need for further research to elucidate the potential benefits and drawbacks of this graft type. Moreover, in some areas, no consensus is reached for graft type recommendations, particularly regarding graft diameter, also in relation to the height of the patient. As more and more research is conducted, this review serves as a guideline, delineating what is known, but equally important, what is not known, and by identifying gaps in recommendations for graft type in ACLR.

A strength of this scoping review is that all recent research conducted in the field of ACLR and the decision-making process of graft selection is included. No limitations were set on the patient population, resulting in a complete overview of the literature for all patient populations in the field of ACLR. Moreover, this review shows the gaps in the literature regarding the decision-making process of ACLR graft type.

However, our scoping review has some limitations. Firstly, while patient characteristics are essential for ACLR graft type selection and optimal recovery, the available literature provides limited evidence for specific patient populations, such as patients with work demanding kneeling activities and graft recommendations for males. However, those studies did result in specific recommendations. Studies involving a more diverse population often did not reveal distinct differences in graft preferences. This highlights the importance of studying specific patient characteristics to derive precise graft recommendations tailored to individual needs. Another limitation is the scarcity of information regarding the QT autograft. Some studies describe QT use as very promising, but the lack of evidence surrounding this graft makes it difficult to make a reliable comparison with BPTB and HT autografts. Then, studies often yield conflicting outcomes, complicating the interpretation of the results of the studies and providing a transparent preference for a graft type. Lastly, no quality assessment was done due to the scoping nature of the review, which is also not required for scoping reviews.

More high-quality research is needed to gain a better and more specific understanding of when to use which graft type and which patient characteristics are most important to consider in the decision-making process for ACLR. Research should be conducted in specific populations to establish clear preferences for these populations, as research in a more varied population does not lead to any apparent results.

## Conclusion

Based on the available literature, the conclusion is that a BPTB graft seems to be the preferred choice in young patients, females, and athletes—especially those engaged in pivoting sports. The HT graft seems to be the preferred choice in less active and older patients, along with those involved in sports where knee extensors are vital. Moreover, surgeon preferences were also of importance for graft selection. Selecting the right graft for an individual can significantly reduce the risk of graft failure, facilitate safe return to sports, and enhance overall quality of life. It is important to recognize that the ideal graft choice varies for each person and the individual patient characteristics should be taken into account in the decision-making process.

## Abbreviations

ACL: Anterior cruciate ligament
ACLR: ACL reconstruction
BPTB: Bone-patellar tendon-bone
HT: Hamstring tendon
QT: Quadriceps tendon
RTS: Return to sport
LET: Lateral extra-articular tenodesis
